# Current News

**Published:** 1894-05

**Authors:** 


### Current News.

### MIDWINTER FAIR DENTAL CONGRESS.

The Midwinter Fair Dental Congress will be held at San Francisco, Cal., June next, from the 11th to the 15th inclusive.

All reputable legal dentists of the nine States and Territories of the Pacific slope, together with those east of the Rockies who wish to join us, are expected and urgently requested to attend this meeting, bringing with them anything and everything that may be of interest to the profession. Come prepared to give rather than to take.

Interesting papers and clinics are promised from leading men both East and West.

A meeting is anticipated that will be second only to that held at Chicago last August.

The work is being carried forward by the following officers and chairmen of committees:

President, Luther A Teague, 10 Geary Street, S. F.; Vice-Presidents, S. E. Knowles, 139 Post Street, S. F.; J. A. W. Lundborg, 219 Geary Street, S. F.; Treasurer, W. A. Knowles, 118 Grant Avenue, S. F.; Secretary, W. Z. King, 1001 Valencia Street, S. F.; Assistant Secretary, C. W. Hibbard, 202 Stockton Street, S. F.; Corresponding Secretary, C. E. Post, 14 Grant Avenue, S. F.

*Chairmen of Committees.*—Executive, S. H. Roberts, 120 McAllister Street; Finance, Harry P. Carlton, Crocker Building; Programme, Walter F. Lewis, 462[2] Thirteenth Street, Oakland; Editorial, J. D. Hodgen, 1005 Sutter Street; Membership, T. N. Iglehart, 713 Sutter Street; Invitation, L. L. Dunbar, 500 Sutter Street; Reception, J. A. W. Lundborg, 219 Geary Street; Local Arrangement, F. C. Pague, 819 Market Street; Dental Club, W. J. Younger, 300 Stockton Street; Advisory, Thomas Morffew, 702 Market Street; Association Day, A. F. Merriman, Jr., 1003[2] Broadway, Oakland; Publication, F. Teague, 21 Powell Street; Transportation, R. H. Cool, 1115 Broadway, Oakland.

Charles E. Post, D.D.S.,
*Corresponding Secretary.*

---

### JOINT MEETING OF THE IOWA AND NEBRASKA STATE DENTAL SOCIETIES.

The annual meeting of the Iowa and Nebraska State Dental Societies will be a joint meeting, held May 1 to 4, inclusive, at Council Bluffs, Iowa, and Omaha, Neb. An interesting programme will be presented. All are cordially invited to attend.

W. C. Davis, Lincoln, Neb.,
*Secretary Nebraska Society.*
F. T. Breene, Iowa City, Iowa,
*Secretary Iowa Society.*

### CHANGE IN THE DATE OF THE NEW JERSEY JULY EXAMINATIONS.

The New Jersey Dental Commission will hold its summer examinations at No. 88 Broad Street, Elizabeth, N. J., commencing on the second Tuesday in July, instead of the third Tuesday, as heretofore.

Applications should be in the hands of the Secretary by June 26.

G. Carleton Brown,
*Secretary.*

---

### RECENT PATENTS.

A list of recent patents reported specially for the International Dental Journal:

512,065.—Box for Tooth-Powder. Warren A. Spalding, New Haven, Conn. Filed July 24, 1893.

512,840.—Dental Mould for Teeth. James R. Phelps, Marysville, Cal. Filed June 20, 1893.

512,841.—Artificial Tooth-Crown. James R. Phelps, Marysville, Cal. Filed August 17, 1893.

512,856.—Artificial Tooth-Crown. Coldwell C. Beebe, Racine, Wis. Filed November 7, 1892.

513,015.—Dental Forceps. Woodbury S. How, Philadelphia, Pa., assignor to the S. S. White Dental Manufacturing Company, same place. Filed November 13, 1893.

513,016.—Teeth-Separator. Woodbury S. How, Philadelphia, Pa., assignor to the S. S. White Dental Manufacturing Company, same place. Filed November 13, 1893.

513,178.—Adjustuble Angle Attachment for Dental Handpieces. Charles L. Furman and George E. Holland, Peoria, Ill. Filed September 8, 1890.

513,214.—Adjustable Dental-Engine Bracket-Arm. Jere. E. Stanton, Boston, Mass. Filed June 19, 1893.

513,328.—Rubber-Dam Clamp. James W. Ivory, Philadelphia, Pa. Filed February 13, 1893.

513,362. Dental Plugger. George A. Foster and Charles E. Hoffman, New Albany, Ind. Filed April 10, 1893.

513,818.—Artificial Tooth. Eli H. Neiman, York, and Samuel E. Beecher, Milton, Pa.; said Beecher assignor to said Neiman. Filed December 29, 1892.

Trade-Marks.—24,149.—Tooth Paste or Dentifrice. William J. Hurd, St. Paul, Minn. Filed November 14, 1893. Essential feature, the words "King Bee" and the representation of a bee's body with a portrait of the registrant for a head to the same.

Patents which expired January 2, 1894. Granted January 2, 1877:

Reissue.—7452.—Dental Drills. G. V. Black, Jacksonville, Ill., assignor, by mesne assignments, to S. S. White, Philadelphia, Pa. Patent No. 117,732, dated August 8, 1871. Filed November 22, 1876.

Patents which expired January 16, 1894. Granted January 16, 1877:

186,234.—Electro-Magnetic Dental Pluggers. J. E. Dexter, New York, N. Y. Filed October 23, 1876.

186,287.—Tooth-Brushes. S. Woolverton, Trenton, N. J. Filed August 15, 1876.

186,307.—Cotton-Holders for Dental Use. T. Cogswell, Boston, Mass., assignor to Codman & Shurtleff, same place. Filed August 26, 1876.

Patents which expired January 23, 1894. Granted January 23, 1877:

186,471.—Tool-Carriers for Dental Engines. J. W. Gilbert, Philadelphia, Pa., assignor to S. S. White, same place. Filed December 11, 1875.

186,504.—Angle Attachments for Dental Engines. E. T. Starr, Philadelphia, Pa., assignor to S. S. White, same place. Filed December 5, 1876.

186,522.—Stamping Dental Plates. C. F. Barnard, Victoria, British Columbia. Filed August 8, 1876.

186,580.—Dental Pluggers. C. King, Pittsburg, Pa. Filed July 24, 1876.

Patents which expired January 30, 1894. Granted January 30, 1877:

186,680.—Dental Pluggers. C. King, Pittsburg, Pa. Filed August 21, 1876.

186,782. Barbers' and Dentists' Chairs. L. M. Angle, Chicago, Ill. Filed November 21, 1876.

186,793.—Cases for Dental Instruments. E. P. Brown, Flushing, N. Y. Filed April 3, 1876.

186,870. Chairs. F. L. Patch, Florence, Mass. Filed July 10, 1876.

186,914.—Machine for Folding Gold-Foil for Dental Purposes. R. S. Williams, New York, N. Y. Filed December 28, 1876.

---

### ANNUAL MEETING OF THE WOMAN'S DENTAL ASSOCIATION.

THE Second Annual Meeting of the Woman's Dental Association of the United States was held at the office of Dr. Mary H. Stillwell, 1300 Arch Street, Philadelphia, Pa., Saturday evening, March 3, 1894.

The following officers were elected for the ensuing year: President, Dr. Anna T. Focht; Vice-President, Dr. Elizabeth A. Davis; Recording Secretary, Dr. Emily W. Wyeth; Corresponding Secretary, Dr. Eliza Yerkes; Treasurer, Dr. Matilda Groth.

*Executive Committee.*—Drs. Mary H. Stillwell, Edith L. Brown, Hannah M. Miller, Martha C. Corkhill, and Maria Lasser.

The Vice-Presidents from representative States were re-elected, except that, in New York, Dr. Alice Ireland takes the place of Dr. Olga Neyman Glücksman.

Dr. Cora J. Little, of Nebraska, Dr. Sara May Townsend, of Colorado, and Dr. Mary Weston, of Missouri, were added to the list. The membership—thirty-two at the close of the first year—now numbers forty-three.

EMILY W. WYETH,
*Recording Secretary.*

3920 FAIRMOUNT AVENUE, PHILADELPHIA, PA.

